# Decreased oocyte quality in patients with endometriosis is closely related to abnormal granulosa cells

**DOI:** 10.3389/fendo.2023.1226687

**Published:** 2023-08-16

**Authors:** Weisen Fan, Zheng Yuan, Muzhen Li, Yingjie Zhang, Fengjuan Nan

**Affiliations:** ^1^ The First Clinical Medical College, Shandong University of Traditional Chinese Medicine, Jinan, Shandong, China; ^2^ Department of Gynecology, Affiliated Hospital of Shandong University of Traditional Chinese Medicine, Jinan, Shandong, China; ^3^ College of Acupuncture and Tuina, Shandong University of Traditional Chinese Medicine, Jinan, Shandong, China

**Keywords:** endometriosis, granulosa cells, infertility, mechanism, oocytes

## Abstract

Infertility and menstrual abnormalities in endometriosis patients are frequently caused by aberrant follicular growth or a reduced ovarian reserve. Endometriosis typically does not directly harm the oocyte, but rather inhibits the function of granulosa cells, resulting in a decrease in oocyte quality. Granulosa cells, as oocyte nanny cells, can regulate meiosis, provide the most basic resources required for oocyte development, and influence ovulation. Endometriosis affects oocyte development and quality by causing granulosa cells apoptosis, inflammation, oxidative stress, steroid synthesis obstacle, and aberrant mitochondrial energy metabolism. These aberrant states frequently interact with one another, however there is currently relatively little research in this field to understand the mechanism of linkage between abnormal states.

## 1 Introduction

Endometriosis (EMS) is a common condition in reproductive-age women. Its most common clinical signs are pelvic pain, dyspareunia, a prolonged menstrual period, and a rise in menstrual volume, which can lead to infertility, anxiety, and depression. Ovarian endometriosis (OEM) has the potential to progress to ovarian cancer (1). It is concerning that the etiology of EMS is unclear and has been debated for a long time. There are numerous contentious causes of EMS at the moment, including endometrial cell implantation hypothesis, body cavity metaplasia theory, induction theory, genetic variables, immune factors, and so on (2). The prevalence of this condition has risen in recent years as contemporary medical diagnosis and treatment technology has advanced. At the moment, the primary method of treatment is surgical intervention and hormone therapy, but it is easy to relapse after surgical treatment, which not only costs more but also causes repeated trauma to the patient, especially when patients with OEM and fertility needs are repeatedly stripped by laparoscopic surgery, which will further damage the patient’s reproductive ability (3, 4). Although hormone is effective in treating this disease, it does not improve the ovarian reserve of EMS patients. As a result, we must first understand how EMS lowers patient fertility, which will allow us to develop effective therapies and preventive measures to assist EMS patients in conceiving.

Reduced ovarian reserve and follicle quality, changes in normal pelvic physical environment, decreased endometrial receptivity, and immunological dysfunction are the main reasons for EMS impeding female fertility. The most concerning aspect is that certain EMS patients are unable to generate qualifying eggs, which reduces the success rate of natural conception and assisted reproduction (5). Despite the fact that the meta-analysis found a minor variation in the success rate of assisted reproduction between patients with EMS and those without EMS. However, there are numerous risk factors for long-term pregnancy in EMS patients (6, 7). Because of the delay in diagnosing EMS, some patients with EMS are clinically diagnosed when their ovarian reserve has been compromised, and this damage is difficult to reverse (8). Oocytes are discharged during the maturity of the follicle, the ovary’s most essential functional unit. Follicular granulosa cells(GCs) are the “guards” that accompany oocytes as they grow in size. During egg cell development, they rely mainly on GCs promotion and communication between oocyte and GCs (9). Recent research has demonstrated that the presence of EMS disrupts energy metabolism, apoptosis, and steroid hormone synthesis in GCs, lowering oocyte quality and limiting patients’ reproductive hopes. As a result, this paper explains how EMS injury lowers oocyte quality by causing follicular GCs damage.

## 2 Ovarian reserve and oocyte quality are reduced in EMS patients

The essence of EMS is that endometrial cells occur in places other than the uterine cavity, with the most common locations being the surface of tissues or organs such as the ovary, utero-rectal pouch, sacral ligament, bladder, and ureter. EMS pathophysiology is comparable to tumor biology in that it involves enhanced proliferation, adhesion, and invasion, increased neovascularization in ectopic endometrial lesions, and decreased apoptosis (10). Endometrial stromal cells from ectopic lesions had significantly increased proliferation, migration, and invasion capacities as compared to normal female endometrial stromal cells, according to research (11). Endometriotic lesions, like cancers, rely on angiogenesis to proliferate. Vascular endothelial growth factor may be produced by the endometrium in the uterine cavity of EMS patients, and the amount of Vascular endothelial growth factor in peritoneal fluid of EMS patients is much higher than that of normal individuals (12). Ectopic endometrial cells respond to estrogen and progesterone in the same way as eutopic endometrial cells do. Surprisingly, some ectopic endometrial cells can also manufacture estrogen on their own (13). Estrogen production is closely related to EMS-associated inflammation. Estrogen can stimulate Cytochrome c oxidase subunit 2 synthesis, and Cytochrome c oxidase subunit 2 can enhance Prostaglandin E2(PGE2) expression. PEG2 can increase the expression of aromatase, which in turn increases estrogen synthesis (14). Estrogen works on the highly expressed estrogen receptors in EMS lesions, promoting endometrial stromal cell survival and invasion, producing pro-inflammatory factors, and perpetuating inflammation (15, 16). The ultimate result of EMS is fibrosis, which is frequently histologically characterized as overly dense fibrous tissue around endometrial glands and stroma (17). Long-term inflammation of endometriotic lesions, which activates the Transforming growth factor beta-1 proprotein(TGF-β) signaling pathway, results in the creation of fibrotic lesions (18). Transforming growth factor beta-1 proprotein levels were observed to be higher in the serum and peritoneal fluid of EMS patients compared to healthy women (19).

Inflammatory, fibrotic, and oxidative responses caused by EMS can all harm a patient’s ovarian reserve. OEM, in particular, can have a direct impact on a patient’s ovarian reserve. In the ovaries afflicted by EMS cysts, the number of primordial follicles and AMH level fell, whereas the number of atretic follicles and primary follicles increased (20). This could be because EMS causes inflammation and oxidative stress, which leads to the recruitment of dormant primordial follicles into the growth and development track, while the local inflammatory response of the foci leads to ovarian fibrosis, affecting ovarian blood supply, and the growing follicles cannot get enough nutritional support and enter the atresia state (21, 22). A vicious cycle ensues, resulting in a patient’s decreasing ovarian reserve and oocyte quality. The most obvious impact is EMS patients have a lower oocyte retrieval rate, a lower oocyte fertilization rate, a lower number of final available embryos and high-quality embryos, and a worse cumulative live birth rate of IVF cycles (23, 24). The ovarian damage of EMS patients is more severe as the disease progresses. According to other research, compared to normal women, the fertilization rate of oocytes in stage I/II EMS patients is around 7% lower, and the fertilization rate in stage III/IV EMS patients is even lower (25). The ultrastructure of EMS patients’ oocytes revealed brown degeneration, deeper cytoplasm, larger refractive body, incomplete protrusion or separation of the first polar body, and a prolonged disintegration time of the zona pellucida (26, 27). Not only is the maturation ability of oocytes from EMS patients reduced, but also cortical granule loss, spindle fragmentation, and zona pellucis sclerosis may develop in immature oocytes following maturation and culture to meiosis II *in vitro*, which may impede with sperm penetration (28).

## 3 GSs’ effect on oocytes

The female reproductive system’s fundamental functioning component is the follicle. Oocytes, GCs, theca cells, and follicular antrum make up the mature follicle. The oocyte is surrounded by flat undifferentiated GCs at the primordial follicle stage. A zona pellucida forms around the oocyte as the follicle develops, separating the GCs. Flat, undifferentiated GCs will become cuboidal and proliferate progressively. Transzonal projections(TZPs) connect the cumulus granulosa cells to the oocyte primarily through the zona pellucida (29). TZPS bind to the oocyte via Gap-junctions (GJs), which allow low-molecular-weight messages or nutrients to be transferred from cell to cell. Cumulus granulosa cells(CGCs) that are distant from the oocyte will form filopodia to interact with the oocyte as it develops (30). Undifferentiated GCs will gradually differentiate into mural granulosa cells(MGCs) and CGCs during the follicle development stage to preantral follicles. MGCs primarily execute endocrine tasks and are the first to accept Follicle stimulating hormone(FSH) and Luteinizing hormone(LH) action.

The function of follicular GCs is to help oocyte development and fertilization. These functions are mostly manifested in four areas (29): 1)Involved in maintaining of oocyte meiotic arrest; 2)Oocyte meiosis was induced to recover; 3)Provide the most fundamental substance needed for oocyte maturation; 4)Influences oocyte ejection from the follicle. Following female birth, the high level of Cathelicidin antimicrobial peptide(cAMP) secreted by undifferentiated GCs can keep cyclin Maturation promoting factor inactive by activating adenylate cyclase in oocytes, causing oocytes to enter the prophase stage of meiosis I (31). Following the appearance of the zona pellucida in oocytes, cyclic guanosine monophosphate(cGMP) secreted by GCs enters the oocyte via GJs, inactivating cGMP-inhibited 3’,5’-cyclic phosphodiesterase 3A and maintaining a high level of cAMP in oocytes that cannot be degraded, thereby inhibiting the meiotic process (32). When women enter the period of sexual development, gonadotropins can act on the follicles, causing them to develop further. When LH secretion was at its height, it might activate mitogen-activated protein kinase3/1(MAPK3/1) in CGCs and decrease the concentration of natriuretic peptide precursor type C in MGCs, resulting in cAMP hydrolysis (33). Activated MAPK also lowered GJs permeability, limiting cAMP and cGMP transport to oocytes and resulting in meiotic recovery of oocytes (34). Furthermore, after receiving the LH signal, the CGCs depolarized, increasing the intracellular calcium ion(Ca2+)concentration (35). Elevated Ca2+ will enter the oocyte via GJs, resulting in a temporary increase in Ca2+ concentration (36). The rise in Ca2+ concentration in oocytes will facilitate meiosis recovery (37).

**Figure 1:**
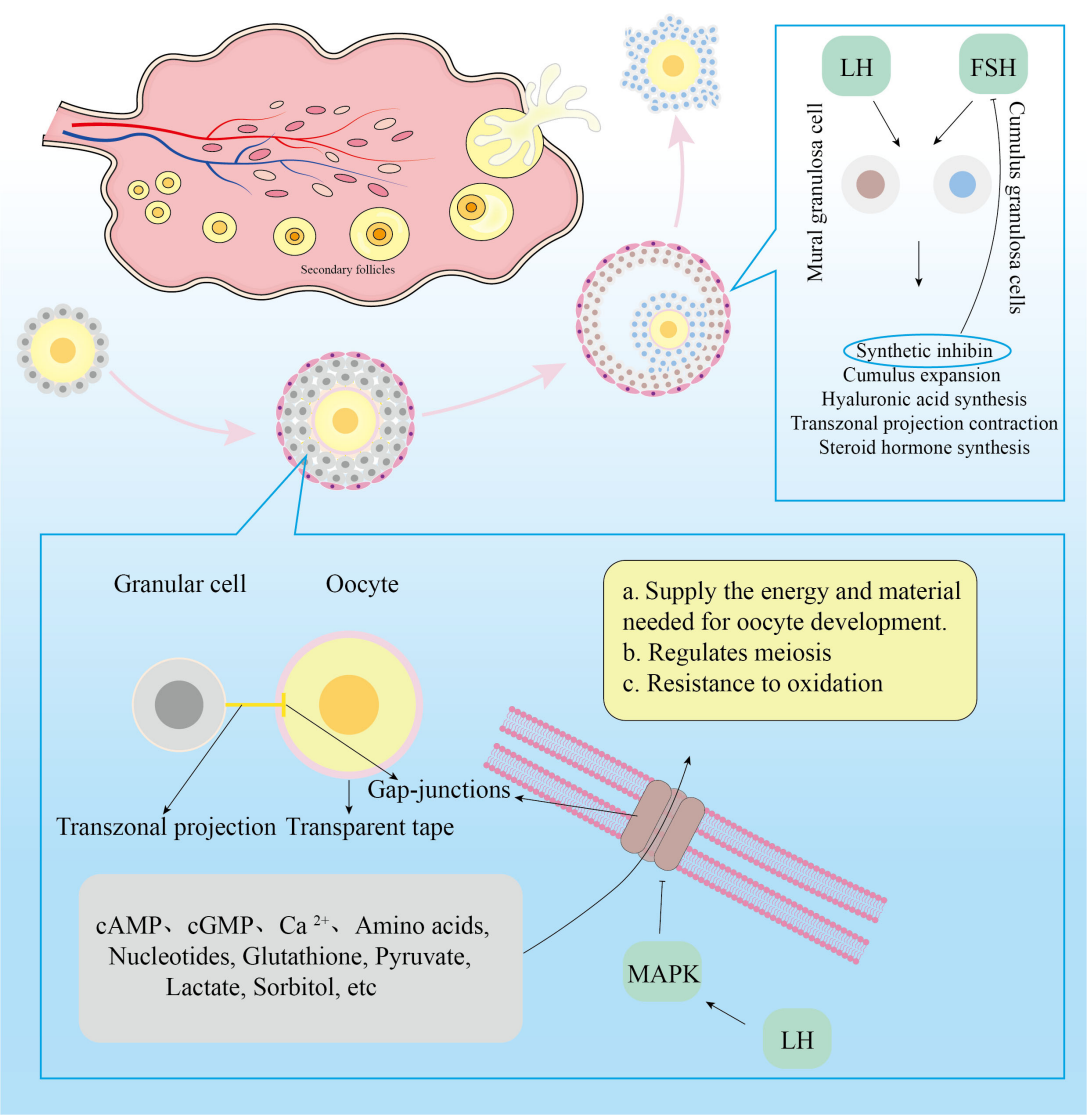
GCsls**’** physiological roles during follicular development.

Because oocytes have a limited ability to utilize nutrients, CGCs provide the majority of them. Through TZPs and GJs, GCs can deliver amino acids, nucleotides, glutathione, and carbohydrate metabolites to oocytes (38). Because of the limited activity of phosphofructokinase and lactate dehydrogenase in oocytes, as well as the sluggish glycolysis process, glucose is primarily glycolyzed by CGCs to create pyruvate and lactate, which are then transported to oocyte mitochondria (39). Oocytes, in turn, can ensure pyruvate availability by increasing the expression of glycolytic genes in CGCs. In addition, sorbitol dehydrogenase in granulosa cells reduces glucose to form sorbitol, which is then fed by oocytes’ indirect fructose synthesis (40). GCs not only provide necessary resources for oocyte formation, but they also control germinal vesicle rupture. FSH and LH control follicle growth, however the oocyte lacks Follicle stimulating hormone receptors (FSHR) and Luteinizing hormone receptors (LHR). As a result, FSH and LH influence follicular growth by acting on FSHR and LHR in CGCs and MGCs. FSH primarily works on MGCs, promoting estrogen synthesis in MGCs as well as GC proliferation. When GCs receive FSH signals, they produce inhibin, which inhibits FSH secretion and promotes the development of dominant and atresia non-dominant follicles (41). FSH and E2 synergistically encourage GCs to create LHR in preparation for the subsequent LH signal. FSH has the ability to stimulate CGC proliferation and hyaluronic acid synthesis. FSH stimulates CGC proliferation, hyaluronic acid synthesis, and then promotes the expansion of cumulus oocyte complex (42). Cumulus oocyte complex quality represents oocyte quality and is required for oocyte maturation and fertilization (43–45). When LH levels rise, LH and FSH work together to promote the production of progesterone and progesterone receptors in CGCs and to withdraw TZP from the oocyte membrane to the CGCs in preparation for the oocyte germinal vesicle to rupture (46, 47). **Figure 1** depicts a more intuitive role for granule cells.

## 4 EMS causes GCs destruction

### 4.1 Apoptosis in GCs was triggered by EMS

When the granulosa cells die, the oocyte suffers from a quality decline or atresia due to a lack of various growth substances required for development. EMS can severely disrupt the cell cycle of follicular granulosa cells and increase the apoptotic rate of granulosa cells in patients (48, 49). Apoptotic bodies are extracellular vesicles containing nuclear and cytoplasmic debris that form as a result of apoptosis. The number of apoptotic bodies in undifferentiated GCs was considerably higher in patients with EMS compared to patients without EMS, and it was higher in patients with OEM compared to patients with EMS at other sites (50). Undifferentiated GC apoptosis can predict the result of *in vitro* fertilization (51). The more apoptotic bodies there are in EMS undifferentiated GCs, the lower the rate of recovered oocytes and the higher the rate of empty follicles.

The targets of differentially expressed CircRNAs were predominantly connected to apoptosis, Phosphatidylinositol3-kinase-RAC-beta serine/threonine-protein kinase(PI3K-AKT), and Cellular tumor antigen p53 signaling pathways by sequencing the CGCs of women with EMS and women without EMS (52). The PI3K-Akt signaling pathway is a critical regulator of cellular transcription, translation, proliferation, growth, and survival. After being phosphorylated, AKT participates in important biological processes such as apoptosis, protein synthesis, and cell cycle progressionAfter being phosphorylated, AKT participates in important biological processes such as apoptosis, protein synthesis, and cell cycle progression. Apoptosis of granulosa cells is higher in patients, which may be associated to decreased serum testosterone levels. In the CV434 granulosa cell line, testosterone can inhibit the PI3K-AKT signaling pathway and reduce cell death (49). Furthermore, the over-activation of primordial follicles in EMS patients’ ovaries is linked to the activation of the PI3K-AKT pathway (53). It was discovered by sequencing the follicular GCs of OEM patients and normal female follicular GCs that the differently expressed genes were primarily abundant in the MAPK, Protein WNT(WNT) signaling pathway, apoptosis, and steroid hormone response (54). Among these, the Wnt signaling pathway is linked to cell proliferation, death, and migration (55). WNT4 and WNT5a transcription levels were significantly increased in luteinizing granulosa cells from EMS patients, while WNT1 transcription levels were significantly decreased, β-catenin and its dephosphorylated active form expression was decreased, and the expression of apoptosis inhibitor genes was decreased, while apoptosis was enhanced. This shows that Wnt signaling dysregulation is linked to granulosa cell death and follicular cell atresia (48).

### 4.2 EMS promotes inflammation and oxidative stress in GCs

Inflammation and oxidative stress can impair oocyte quality and potentially cause follicular atresia. EMS, especially OEM, can lead to follicular inflammation and oxidative stress. According to previous research (56), the levels of C-C motif chemokine 2(CCL2) and Interleukin(IL)-8 in the follicular fluid produced by EMS-affected ovaries are higher than those produced by normal ovaries. CCL2 is a tiny cytokine that has the ability to cause inflammation. It can not only attract inflammatory cells like neutrophils, monocytes, and lymphocytes to the lesion site, but it can also stimulate the production of additional cytokines including IL-2, IL-6, and cell adhesion molecules (57). IL-8 is also a chemokine cytokine, and its involvement and regulation of human reproductive physiological and pathological processes has been established. IL-8’s primary biological activity is to recruit and activate neutrophils, hence promoting inflammation (58). The inflammatory reaction involving the follicles may become more severe as the lesions progress. IL-23 levels in follicular fluid and serum, for example, were considerably greater in individuals with III-IV EMS compared to those with I-II stages (59). In autoimmune inflammatory illnesses, IL-23 is a related factor that can mediate inflammatory and immunological responses by T cells, NK cells, and macrophages (60). When granulosa cells from EMS patients were cultivated *in vitro*, the levels of Tumor necrosis factor ligand superfamily member(TNF)-α, IL-8, and IL-1β in the cell supernatant were greater than in the control group (61). Furthermore, Nuclear Factor Kappa B(NFκB), Inhibitor of nuclear factor kappa-B kinase subunit beta, and NF-kappa-B inhibitor alpha expression was increased in granulosa cells from ovarian EMS patients, and the NFκB signaling pathway was significantly activated when granulosa cells were cultured with TNF-α (62). NFκB is an essential regulator of cellular inflammatory response and is involved in increasing the inflammatory cascade through cytokine activation. Telomerase activity is high in healthy follicles, whereas NF-kB expression in granulosa cells is inversely associated to oocyte mass and telomerase activity, implying that post-inflammatory alterations in granulosa cells are deleterious to oocyte development (63). However, Liang found no significant differences in chemokines and inflammatory cytokines between patients with surgically removed endometrial cysts and those with untreated EMS in follicular fluid (64).

Inflammation and oxidative stress are interrelated, and increases in oxidative stress can cause acute and chronic inflammation (65). Oxidative stress can cause inflammation via a number of mechanisms, including Nucleotide-binding oligomerization domain-like receptors, TOLL receptors, and NFκB pathways (66). Simultaneously, inflammation can result in oxidative stress (67, 68). The oxidative stress level in the ovarian cortex around endometriotic cysts was much higher than in dermoid cysts (69). The composition of follicular fluid revealed that patients with EMS had higher levels of oxidative substances such as 8-hydroxy-2 deoxyguanosine, reactive oxygen species, peroxynitrite ion, Nitric oxide, and malondialdehyde, and lower levels of antioxidant substances such as peroxide dismutase, catalase, vitamin A, vitamin C, vitamin E, and reduced glutathione (70–72). This could be connected to EMS-induced senescence, endoplasmic reticulum stress, and oxidative stress in CGCs (73, 74). When mouse cumulus oocyte complexes were cultured with endometrial cyst fluid, CGC mitochondrial performance was impaired, glutathione content was reduced, reactive oxygen species levels rose, and oxidative damage to oocytes was hastened (75). This shows that EMS can impact oocyte quality by generating oxidative stress in GCs. However, Donabela’s study found that the expression of superoxide dismutase 1, an antioxidant, was elevated in CGCs from individuals with moderate-to-severe EMS (76). As a result, we cannot say if the presence of EMS causes adaptive changes in CGCs, such as increased antioxidant capability. Oxidative stress may hinder ovulation in addition to lowering oocyte quality. Lin discovered that oxidative stress might decrease the expression of the histone-lysine N-methyltransferase EZH2 and the level of lysine 27 of the histone H3 protein in GCs while increasing the expression of Interleukin-1 receptor type 2 to suppress ovulation signals (77).

### 4.3 EMS influences GCs steroid hormone production

The synthesis and secretion of steroid hormones by GCs is critical for follicular growth and, as a result, can impact the quality of oocytes. The amount of FSHR and LHR on the surface of MGCs steadily increases as follicles expand, and estrogen released by MGCs can enhance CGCs proliferation. The aberrant follicular development in EMS patients is linked to a defective FSH signaling pathway operating on GCs, and EMS patients respond to FSH less effectively during ovulation induction (78). Although FSHR and cytochrome P450 family 19 subfamily A member 1(CYP19A1) expression levels are lower in GCs from EMS patients (77), it is unknown how EMS specifically changes FSHR signaling. According to other research (79, 80), the levels of estrogen and testosterone in the follicular fluid of patients with EMS are lower than those of patients without EMS, but the amount of progesterone is higher. When estrogen levels in the follicle are low, it frequently represents a deterioration in oocyte quality and the failure of *in vitro* fertilization (81). Follicular fluid progesterone levels rise as EMS severity rises, whereas testosterone levels fall as EMS severity rises (82, 83). However, another investigation found no change in progesterone synthesis by granulosa-lutein cells between patients with and without EMS (84). As a result, more information is required to establish the status of steroid synthesis by GCs in EMS.

The GCs produce and secrete the majority of the hormones in the follicle, and the hormone level in the follicular fluid represents the GCs’ steroid hormone secretion ability. The increased progesterone release by GCs may be related to increased autophagy of GCs in EMS patients, higher expression of Beclin-1(BECN1), and greater low-density lipoprotein degradation capacity. BECN1 inhibition decreases GCs autophagy and lowers low-density lipoprotein-induced progesterone synthesis (85). Furthermore, unusually increased PGE2 levels in EMS patients’ follicular fluid can enhance the expression of Steroidogenic acute regulatory protein(StAR) and the synthesis of progesterone in GCs (78). STAR may transport cholesterol from the outside mitochondrial membrane to the inner mitochondrial membrane and convert cholesterol to pregnenolone. Pregnenolone is a progestogen precursor that causes progesterone to be produced in response to 3β-HSD. However, Sreerangaraja’s (79) study found that the expression of STAR and 3β-HSD was reduced in CGCs from EMS patients. As a result, the mechanism by which EMS influences progesterone production by granulosa cells is unclear and requires additional investigation.

GCs from EMS patients exhibited lower expression of not just 3β-HSD, but also CYP19, as well as impaired ability to release inhibin B and estrogen (79, 86, 87). Among them, 3β-HSD was capable of converting dehydroepiandrosterone to androstenediol.CYP19 catalyzes the conversion of androstenedione and testosterone into estrone and estradiol, respectively, and is the rate-limiting enzyme in estrogen biosynthesis. Both proteins are essential regulators of the estrogen synthesis process and have a rate-limiting effect on the synthesis of steroid hormones, which may explain why GCs produce less estrogen. The mechanism through which EMS interferes with estrogen synthesis by GCs is currently unknown. A reliable theory is that the presence of EMS activates the extracellular regulated protein kinase(ERK)1/2 signaling pathway in GCs. When the ERK1/2 signaling pathway is activated, it inhibits estrogen production by CYP19 and promotes progesterone generation by STAR (88). Elevated IL-6 levels in EMS patients’ follicular fluid may activate the ERK1/2 signaling pathway in GCs. When Deura utilized IL-6 to culture granulosa tumor cell lines, it could enhance ERK1/2 phosphorylation, limit CYP19 expression, and diminish estrogen release (89). Li’s research also discovered that ERK1/2 signaling is enhanced in granulosa cells from EMS patients (90).

### 4.4 EMS influences mitochondrial energy metabolism in GCs

Because GCs are the nanny cells that feed the oocyte, mitochondria play a vital role in energy metabolism. Therefore, mitochondrial morphology, the amount of mitochondrial DNA(mtDNA) expression, and the efficiency of adenosine-triphosphate(ATP) synthesis in GCs all influence oocyte developmental potential to some extent (91). Mitochondria from CGCs from mild EMS patients showed morphological edema, hazy mitochondria, and reduced mtDNA expression abundance (92). After surgery, the quantity of mtDNA expression in follicular GCs of females with severe EMS was enhanced compared to women without EMS (93). This could be related to a compensatory increase in mtDNA expression in GCs to compensate for the ovary’s lack of mitochondrial energy metabolism in order to adapt to the hypoxia in the follicular development microenvironment. According to Hsu’s research (94), CGCs in EMS patients produced less ATP, although mtDNA expression abundance remained unaffected. The respiratory chain’s structural proteins are encoded by mtDNA. The loss of its nucleic acid sequence will hamper oxidative phosphorylation and decrease ATP generation. As a result, when the energy metabolism of GCs mitochondria is insufficient, oocyte quality suffers. EMS also decreases mitochondrial membrane potential in GCs (79). The stability of mitochondrial membrane potential promotes the preservation of normal cellular physiological function. Normal mitochondrial membrane potential is required for oxidative phosphorylation and the generation of ATP. Increased glucose intake and lactate generation are also signs of abnormal mitochondrial energy metabolism in GCs from EMS patients. This could be due to elevated Prohibitin 1(PHB1) expression in GCs of EMS patients. When PHB1 expression was reduced, GCs expression of enzymes involved in glucose metabolism, glucose consumption, and lactate generation decreased (95). Furthermore, Sirtuin 2(SIRT2) inhibits phosphoenolpyruvate carboxykinase 1 degradation, and Phosphoenolpyruvate carboxykinase, cytosolic [GTP] is the rate-limiting enzyme in gluconeogenesis. SIRT2 expression is enhanced in granulosa cells of EMS patients, indicating that EMS impacts GCs metabolic pathways, which may be mediated by SIRT2 (96).

## 5 The relationship between EMS-induced GCs abnormalities and oocyte quality

**Figure 2:**
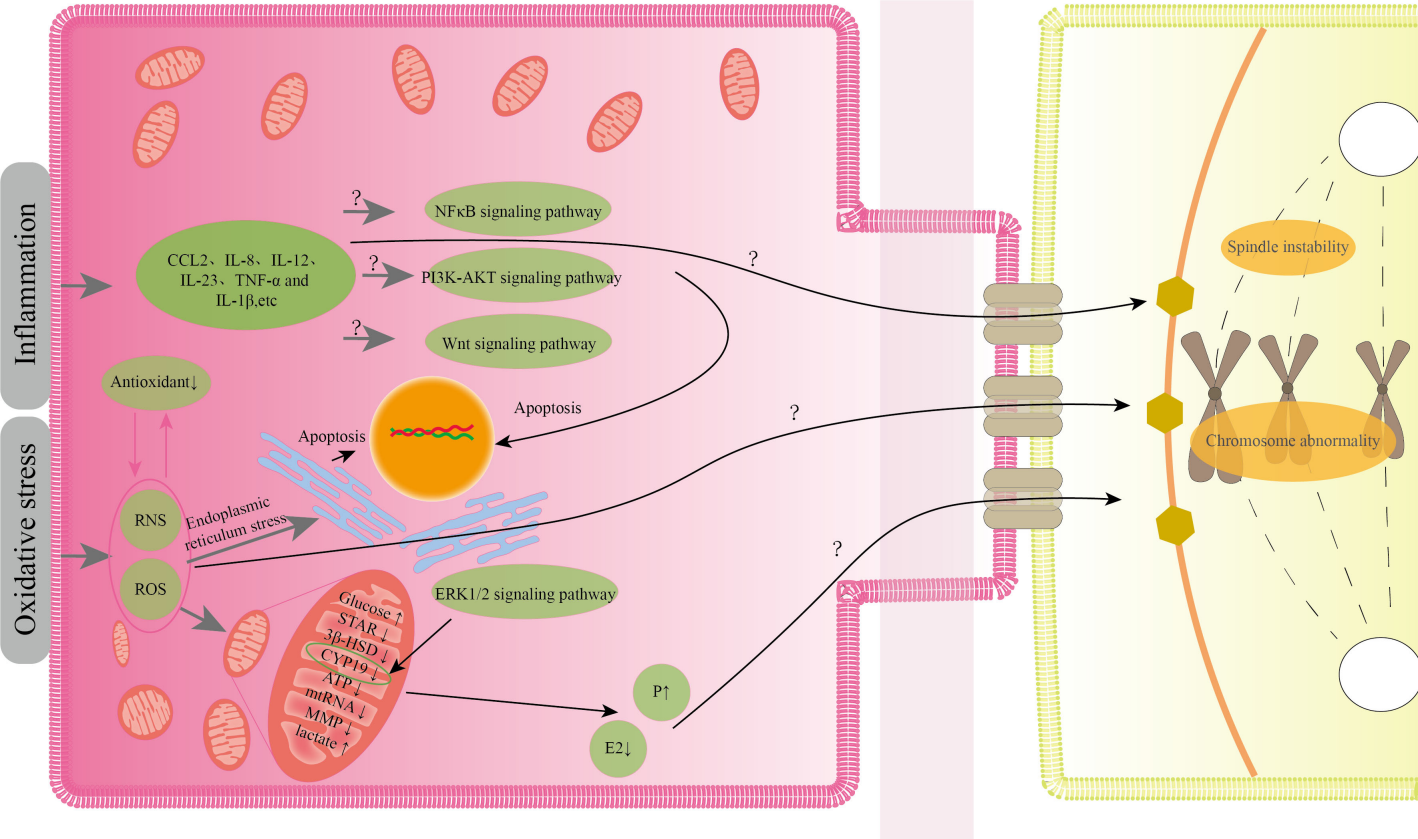
Possible ways by which the aberrant condition of granulosa cells impacts oocyte quality.

Current research indicates that EMS-induced oxidative stress, inflammation, aberrant mitochondrial energy metabolism, inappropriate steroid production, and apoptosis in GCs can all impair the quality of oocytes to variable degrees. Although there have been few studies on the abnormal pathological states of GCs, it is known from the existing studies that the abnormal condition of GCs generated by EMS does not exist alone, but interacts with one another. A transcriptome analysis of CGCs revealed that the genes that differed between mature and immature CGCs were mostly involved in steroid metabolism, inflammation, apoptosis, cell cycle regulation, and extracellular matrix remodeling (97). This also implies that the aberrant status of GCs generated by EMS will have an effect on oocyte maturation. According to the current research, oxidative stress appears to be the root cause of various aberrant conditions. EMS-induced oxidative stress generates reactive oxygen species, and an increase in reactive oxygen species in the follicle leads to spindle instability, aberrant chromosomal formation, and impaired oocyte developmental capability (98). Furthermore, protein nitration is enhanced in follicular GCs of EMS patients, and protein nitration is a marker of peroxynitrite ions, which are reactive nitrogen free radicals (70). Peroxynitrite ions can change oocyte spindle shape and chromosomal organization in a dose-dependent way (99). Oxidative stress can damage GCs mitochondria, affecting mitochondrial energy metabolism and steroid hormone production. Mitochondrial dysfunction generates free radicals, which exacerbates oxidative stress (100). Furthermore, the lower mitochondrial membrane potential of GCs may cause nuclear and cytoplasmic maturation to be out of sync, eventually leading to embryo development failure (101). The failure of mitochondrial steroidogenesis in GCs can result in aberrant oocyte development, especially when estrogen levels in follicular fluid are low and progesterone levels are high, affecting proper meiosis and late cleavage of oocytes (80, 102, 103). Nevertheless, it is unknown whether the level of progesterone in follicular fluid of patients with EMS differs from that of normal individuals, and we do not yet know the mechanism by which progesterone influences oocyte quality. Oxidative stress can also cause GCs inflammation and apoptosis. GCs inflammation frequently interferes with oocyte meiotic capacity. Lipopolysaccharide-induced inflammation enhances IL-6 and IL-8 secretion in bovine GCs, resulting in meiotic block and failure of germinal vesicle rupture in oocytes (104). The IL-6 described above may interfere with estrogen synthesis in GCs, impacting oocyte development (88). Several investigations in embryo culture have demonstrated that IL-8, IL-12, and TNF-α in EMS patients’ follicular fluid are inversely connected with oocyte maturity and embryo quality (105). Among these, TNF-α production by CGCs can increase senescence of mouse oocytes after ovulation, which may be the cause of oocyte quality decline (106). Although the mechanism of EMS-induced granulosa cell death is unknown, oxidative stress-induced apoptosis could be produced by endoplasmic reticulum stress. When EMS follicles were compared to normal women, GCs apoptosis was enhanced and endoplasmic reticulum stress was visible. GCs apoptosis was reduced after endoplasmic reticulum stress was relieved (73). MtDNA depletion causes apoptosis in granulosa cells (107). We noted previously that EMS patients’ granulosa cells had abnormal mtDNA expression, but we don’t know how EMS influences the abnormal expression of mtRNA. **Figure 2** illustrates a potential process by which the granulosa cells' aberrant condition influences the quality of the oocyte.

## 6 Summary

The decrease in oocyte quality in EMS patients will make pregnancy hard for women. Even with assisted reproductive technology, the rate of pregnancy failure remains high. In conclusion, EMS primarily reduces oocyte quality by causing GCs apoptosis, inflammation, oxidative stress, steroidogenesis disorders, and abnormal mitochondrial energy metabolism, which also provides a therapeutic direction for improving assisted reproductive technology success rates in EMS patients. It is troubling that these aberrant states frequently affect each other, but the mechanism underlying the link between the multiple abnormal states of GCs generated by EMS is yet unknown. In addition, it is unclear how the aberrant status of GCs influences the oocyte by which mechanism or pathway. Although patients with EMS can achieve pregnancy using assisted reproductive technology, it is unknown whether the offspring’s health would suffer as a result of poor oocyte quality. In terms of research technology, current study on the extraction of GCs from follicles leaves key questions unanswered. Because MGCs and CGCs serve different functions in follicles, most studies do not distinguish between the two when removing GCs from follicles. As a result of our summary, future research should concentrate on how to improve the abnormal condition of GCs induced by EMS and better understand the routes by which the abnormal state of GCs impacts oocyte quality. We can intervene earlier to achieve better GCs, enhance reproductive endocrinology, and so increase pregnancy rates and offspring health if we understand how EMS affects oocyte quality by altering GCs.

## Author contributions

Writing—Original draft preparation: WF and ZY. Writing—review and editing, and supervision: ML and YZ. Draw diagram: WF. All authors contributed to the article and approved the submitted version.
